# WD40 Domain Divergence Is Important for Functional Differences between the Fission Yeast Tup11 and Tup12 Co-Repressor Proteins

**DOI:** 10.1371/journal.pone.0011009

**Published:** 2010-06-08

**Authors:** Monica E. Ferreira, Kurt D. Berndt, Johan Nilsson, Anthony P. H. Wright

**Affiliations:** 1 School of Life Sciences, Södertörn University, Huddinge, Sweden; 2 Department of Laboratory Medicine, Karolinska Institutet, Huddinge, Sweden; 3 Center for Biosciences, Department of Biosciences and Nutrition, Karolinska Institutet, Huddinge, Sweden; University of Washington, United States of America

## Abstract

We have previously demonstrated that subsets of Ssn6/Tup target genes have distinct requirements for the *Schizosaccharomyces pombe* homologs of the Tup1/Groucho/TLE co-repressor proteins, Tup11 and Tup12. The very high level of divergence in the histone interacting repression domains of the two proteins suggested that determinants distinguishing Tup11 and Tup12 might be located in this domain. Here we have combined phylogenetic and structural analysis as well as phenotypic characterization, under stress conditions that specifically require Tup12, to identify and characterize the domains involved in Tup12-specific action. The results indicate that divergence in the repression domain is not generally relevant for Tup12-specific function. Instead, we show that the more highly conserved C-terminal WD40 repeat domain of Tup12 is important for Tup12-specific function. Surface amino acid residues specific for the WD40 repeat domain of Tup12 proteins in different fission yeasts are clustered in blade 3 of the propeller-like structure that is characteristic of WD40 repeat domains. The Tup11 and Tup12 proteins in fission yeasts thus provide an excellent model system for studying the functional divergence of WD40 repeat domains.

## Introduction

The *Saccharomyces cerevisiae* Ssn6/Tup1 complex has been extensively studied and serves as a model co-repressor that is required for transcriptional regulation of a variety of genes, including genes involved in mating, stress and metabolic pathways [1]–[3]. The metazoan Gro/TLE proteins are functional homologs of Tup1 that are encoded by several genes, some of which are alternatively spliced to create further variety within the family of co-repressor proteins. Gro/TLE proteins regulate many processes such as embryonic development and they are also important in the context of adult human disease [4]–[6].

The budding yeast Ssn6/Tup1 complex consists of one copy of the Ssn6 protein and four copies of Tup1, which in turn consists of three functional domains. The N-terminal domain of Tup1 mediates oligomerization and interaction with Ssn6, putatively through insertion of the N-terminal helix bundle into a cavity in Ssn6 [7]–[9]. The middle region of the protein is required for its repressor function and interaction with the N-terminal tails of histones, and the WD40 repeat domain, found in the C-terminal region, forms a seven-bladed propeller-like structure that constitutes a highly conserved protein-protein interaction domain found in various classes of proteins in evolutionary distant organisms [10]–[12]. The Ssn6/Tup1 co-repressor complex does not have intrinsic DNA binding properties but associates with target genes through interaction with DNA-binding transcription factors. It subsequently interacts preferentially with the hypo-acetylated N-termini of histones and represses transcription through changes in nucleosome positioning, interaction with components of the Mediator complex and recruitment of factors involved in repression, such as histone deacetylases (HDACs) [13]–[17].

In the fission yeast *Schizosaccharomyces pombe*, gene duplication has resulted in two genes encoding Tup1 homologs – *tup11^+^* and *tup12^+^*. We have previously shown that *tup11^+^* and *tup12^+^* play distinct roles under some conditions, such as CaCl_2_ mediated stress [18]. However, the mechanistic basis or the functional differences between the Tup11 or Tup12 proteins is not known. Under normal growth conditions on rich media, Tup11 and Tup12 interact with each other and can interact with Ssn6 independently of each other [18]. Under the same conditions, the Tup11, Tup12 and Ssn6 co-localize on chromatin throughout the genome, even on genes for which they play differential roles [19]. The ability to affect target genes differentially in context of a common complex might involve the histone binding properties of the repression domain. The conservation between *S. pombe* Tup11 and Tup12 within this region is even lower than the conservation between either protein and their Tup1 homologues in a range of distantly related fungal species [18]. We hypothesized that the high degree of variation in this region of the proteins might be the result of positive adaptive selection, leading to repression domains with differential affinities for particular histone tails and/or their post-translationally modified versions. Here we use phylogenetic and functional approaches to investigated which regions of Tup11 and Tup12 underlie their divergent functions.

## Results

### The *tup11^+^* and *tup12^+^* genes are conserved in fission yeasts

**Figure 1:**
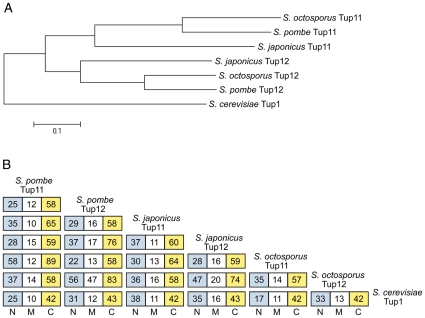
Phylogenetic relationships between Tup11 and Tup12 proteins in fission yeasts. (A) Dendrogram of Tup11 and Tup12 proteins from different fission yeast species, based on ClustalW alignment of full-length translated protein sequences. *S. cerevisiae* Tup1 is used as the outgroup. (B) Cross comparison of similarity between N-terminal domains (N), middle regions (M) and C-terminal domains (C) of budding and fission yeast Tup proteins. The numbers in each box correspond to in the percentage identity of amino acid residues for each pair wise comparison of aligned protein domains. The following protein regions were compared: *S. cerevisiae* Tup1 residues 1–89 (N), 90–317 (M), 318–713 (C), *S. pombe* Tup11 residues 1–87 (N), 88–286 (M), 287–614 (C), *S. octosporus* Tup11 residues 1–87 (N), 88–271 (M), 272–601 (C), *S. japonicus* Tup11 residues 1–87 (N), 88–303 (M), 304–630 (C), *S. pombe* Tup12 residues 1–104 (N), 105–259 (M), 260–586 (C), *S. octosporus* Tup12 residues 1–88 (N), 89–228 (M) 229–555 (C), *S. japonicus* Tup12 residues 1–88 (N), 89–249 (M), 250–576 (C).

Genome sequencing of the fission yeasts, *S. japonicus* and *S. octosporus* [GenBank: AATM01000000, ABHY02000000], allowed us to show that the duplication that gave rise to *tup11^+^* and *tup12^+^* in *S. pombe* is conserved in other fission yeasts. To determine the gene structure of the genes as well as whether they are expressed, we RT-PCR amplified and cloned cDNA corresponding to *tup11^+^* and *tup12^+^* from both *S. japonicus* and *S. octosporus* using primers derived from the respective genome sequences. The expression of both genes in these fission yeast species suggests that the two genes may have diverged functions as has been shown previously for *S. pombe*. Phylogenetic analysis of full-length protein sequences derived by translation of the cDNA sequence showed that the sequences sort into two clades equivalent to *S. pombe* Tup11 and Tup12 (Fig. 1A). The topology of the clades, showing the closest relationship between the *S. pombe* and *S. octosporus* sequences, reflects the species phylogenetic tree derived from analysis of mitochondrial DNA sequences [23].

To test whether the divergent central repression domain might be responsible for the functional difference between Tup11 and Tup12 as suggested previously [18], we compared the relative sequence identity within the N-terminal (N), Middle (M) and C-terminal (C) domains. If sequence variation in the M domain accounts for the functional difference between Tup11 and Tup12 we would expect to see much greater M domain sequence conservation within the groups of Tup11 and Tup12 proteins than between them. Fig. 1B shows that The M domains of Tup12 proteins tend to be more similar to each other than to Tup11 M domains but that no such trend is seen for Tup11 proteins. This would be consistent with the acquisition of a common Tup12-specific function but the M domain of Tup11 seems rather to have accumulated a high level of neutral variation of little or no significance for distinction between Tup11 and Tup12. The domain that most clearly fulfills the above expectation is the C domain that is clearly more conserved within the Tup11 and Tup12 groups than between them. The N domain is intermediate. In summary, phylogenetic analysis gives strongest support to the C-terminal WD40 repeat domain as a determinant of divergent Tup11 and Tup12 functions.

### *In vivo* functions of Tup12 are partly conserved between species

**Figure 2:**
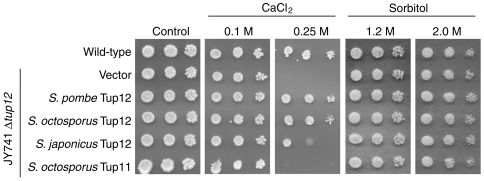
Ability of *S. octosporus* and *S. japonicus* Tup12 proteins to rescue the CaCl_2_ sensitivity of *tup12Δ* in *S. pombe*. Five-fold dilutions of the wild-type strain JY741 and JY741 *Δtup12* transformed with empty vector or with plasmids expressing one of the fission yeast Tup12 proteins or *S. octosporus* Tup11, spotted on YES agar supplemented with CaCl_2_ (0.1 M or 0.25 M, left panels) or sorbitol (1.2 M or 2.0 M, right panels).

The interpretation of the phylogenetic analysis assumes conservation of Tup11 and particularly Tup12 functions in the different fission yeasts. To test this we tested the ability of *tup12^+^* from both *S. japonicus* and *S. octosporus* to complement the CaCl_2_ sensitivity phenotype associated with *S. pombe* strains lacking Tup12. *tup11^+^* from *S. octosporus* was also tested as a negative control. Fig. 2 (left panels) shows that *tup12^+^* from *S. octosporus* complements the Tup12 defect on CaCl_2_ similarly to *S. pombe tup12^+^* while *tup12^+^* from *S. japonicus* complements less efficiently. As expected, *S. octosporus tup11^+^* does not complement at all and none of the strains are sensitive to lower concentrations of CaCl_2_ (0.1 M). We conclude that Tup12 has a conserved role in the response of fission yeasts to CaCl_2_ stress but that this is most clearly conserved between the *S. pombe* and *S. octosporus*, which are more closely related. No growth defects were observed on sorbitol plates (Fig. 2, right panels), demonstrating that the CaCl_2_ sensitivity phenotype is specific for CaCl_2_ and not just due to general osmotic sensitivity.

### The C-terminal domain of Tup12 contains important determinants of Tup12 specific function during CaCl_2_ stress

**Figure 3:**
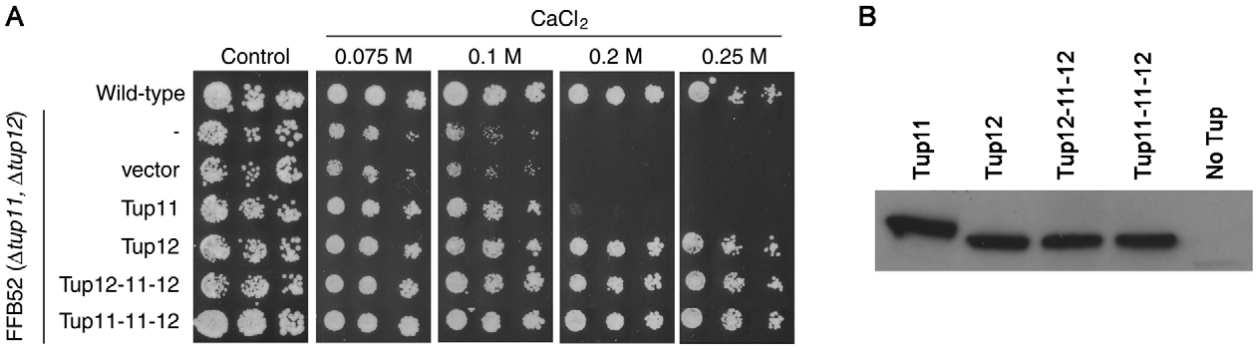
Ability of Tup11/12 hybrid proteins to rescue the CaCl_2_ sensitivity of *tup11*Δ, *tup12*Δ in *S. pombe*. Five-fold dilutions of the wild-type strain JY741, strain FFB52 (*Δtup12*, *Δtup12*) and strain FFB52 transformed with empty vector or with plasmids expressing of intact Tup11 or Tup12 or Tup11/12 hybrid proteins, spotted on YES agar supplemented with increasing concentrations of CaCl_2_. The Tup11/12 hybrid proteins contained the following amino acid residues from Tup11 and Tup12. Tup12-11-12 (Tup12 residues 1–104, Tup11 residues 88–286, Tup12 residues 260–586 followed by Gly, Ser derived from the vector); Tup12-11-11 (Tup12 residues 1–104, Tup11 residues 88–614 followed by Gly, Ser derived from the vector), Tup11-12-12 (Tup11 residues 1–87, Tup12 residues 105–586 followed by Gly, Ser derived from the vector). (B) The hybrid Tup11/12 proteins are expressed at similar levels to Tup11 and Tup12. Western blot in which Flag-tagged proteins were identified using antibodies specific for the Flag tag. The Tup proteins are the same as in A. FFB52 was used as a negative control (No Tup).

To investigate directly whether the M domain is sufficient to account for the functional differences between Tup12 and Tup11 during CaCl_2_ stress, we constructed a *S. pombe* Tup11/12 hybrid protein in which the M domain of Tup12 was replaced by the corresponding domain of Tup11. The hybrid protein was expressed in a *S. pombe* strain lacking both *tup11^+^* and *tup12^+^* to allow testing of the overall functionality of the hybrid protein as well as its Tup12-specific functionality. Fig. 3A shows that the hybrid protein (Tup12-11-12) is generally functional because it can efficiently rescue the low-level CaCl_2_ sensitivity (≤0.1M) that can be rescued by expression of either Tup11 or Tup12. Importantly however, the hybrid can also rescue the high-level CaCl_2_ sensitivity (≥0.2M) that can be rescued by expression of Tup12 but not Tup11. The expression level of the hybrid protein is similar to that measured for Tup12 (Fig. 3B). Therefore, there is no support for the hypothesis that the divergent M domain accounts for Tup12 specific activity during CaCl_2_ stress. Since the phylogenetic analysis (Figs. 1 and 2) pointed to an important Tup12-specific role of the C-terminal domain, we made a hybrid construct to test this directly. The new hybrid protein in which the C-terminal domain of Tup11 was replaced by the corresponding domain from Tup12 (Tup11-11-12) rescues the sensitivity phenotype similarly to Tup12 on both lower and higher concentrations of CaCl_2_. The expression level of this hybrid protein is also similar to that seen for Tup12 (Fig. 3B). We conclude that the C-terminal domain of Tup12 contains important Tup12 specific determinants required for CaCl_2_ stress response.

### Sequence comparison predicts differences in localized surface properties of Tup12 compared to Tup11

**Figure 4:**
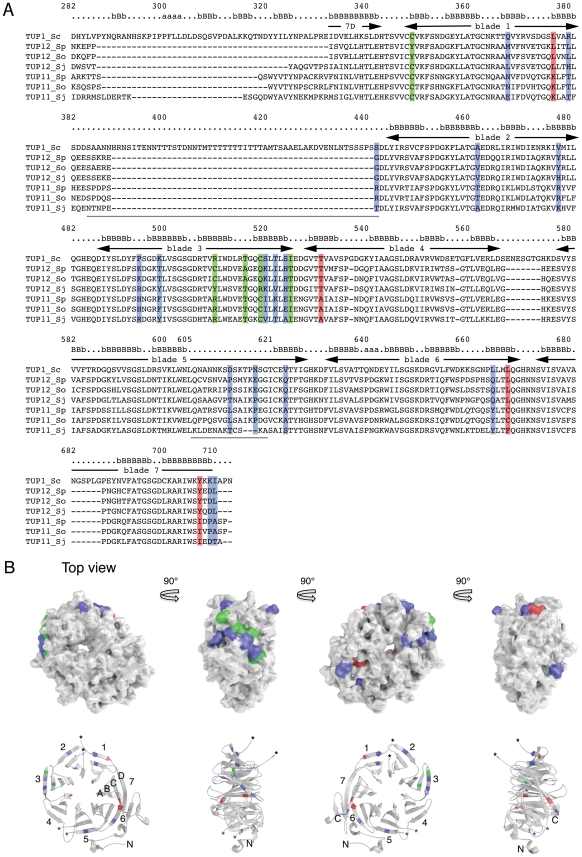
Location of divergent residues in the C-terminal domain that distinguish Tup11 and Tup12. (A) Multiple alignment of the WD40 repeat domains of fission yeast Tup11 and Tup12 proteins in relation to the Tup1 from *S. cerevisiae*. Residues that are identical in the Tup11 or Tup12 proteins from *S. pombe* and *S. octosporus* but which differ significantly between Tup11 and Tup12 are coloured (blue – both Tup11 and Tup12 residues differ from Tup1; red – Tup11 residues are the same as in Tup1; green - Tup12 residues are the same as in Tup1). Secondary structure and numbering of the WD40 domain blades is indicated above the sequence. The amino acid numbering, secondary structure annotation and the position of the so-called blade structures refer to positions in the intact *S. cerevisiae* Tup1 protein. Sequences underlined in grey correspond to disordered sequences that are not seen in the crystal structure (Fig 4B). The sequence residues aligned by ClustalX are *S. cerevisiae* Tup1 (282–713), *S. pombe* Tup11 and Tup12 (282–614 and 271–586), *S. octosporus* Tup11 and Tup12 (271–601 and 240–555) and *S. japonicus* Tup11 and Tup12 (293–630 and 253–576). (B) Tertiary structure of Tup1 showing the location of residues that differ significantly between Tup11 and Tup12 proteins. Colouring is as described in (A) above. The upper and lower panels show space-filling and ribbon diagrams of the WD40 repeat domain, respectively. Different views of the structure were generated by 90° rotations about a verticle axis through the center of mass of the molecule. The N- and C-terminal ends as well as the blades and their component strands are labeled according to convention. Asterisks show gaps in the crystal structure due to the existence of disordered regions. Blue asterisks indicate that the disordered region contains residues that are significantly diverged between Tup11 and Tup12.

Both phylogenetic and functional evidence suggests that the C-terminus is a main determinant of the functional differences that distinguish Tup12 from Tup11, even though it is the most conserved domain between all Tup proteins. To identify differences in the properties of the Tup12 and Tup11 C-terminal domains, we first identified residues that were identical between *S. pombe* and *S. octosporus* but that that were consistently different between the Tup11 and Tup12 proteins of these species. These species were chosen because both have Tup12 proteins that efficiently rescue the CaCl_2_ sensitivity of *S. pombe tup12Δ* mutant cells. Pairs of residues that differ between Tup11 and Tup12 were judged to have significantly different properties if they have a negative score in a BLOSUM62 matrix or if they have opposite net charge. The ClustalX alignment of Tup11 and Tup12 C-terminal regions with secondary structure annotations based on the tertiary structure of *S. cerevisiae* Tup1 [21] is shown in Fig. 4A. The positions of the divergent residues that match the selection criteria are coloured (different colours show the identity of either or neither of the fission yeast proteins with Tup1). The divergent residues that distinguish Tup11 and Tup12 are clearly non-randomly distributed throughout the primary sequence, with a major cluster of divergent residues being located in the region that forms blade 3 of the WD40 propeller-like structure. We used the tertiary structure of the Tup1 C-terminal domain [21] to predict the location of divergent residues that distinguish Tup11 and Tup12 on the surface of the WD40 propeller-like structure. Fig. 4B (upper panels) shows that the majority of the divergent residues are clustered on the side of the structure, in a region corresponding to blade 3 in the WD40 domain propeller structure, shown in Fig. 4B (lower panels). In contrast, the conserved top region, which is known to be important for Tup1 dependent transcriptional regulation [1], [21], [24], is also highly conserved between the Tup11 and Tup12 proteins of fission yeasts (Fig. 4B, left panels). Our data thus suggest a model whereby Tup11 and Tup12 in fission yeasts repress transcription via common mechanisms that are also conserved with Tup1 proteins in more distant species. Tup12 specificity, involving specific amino acid differences in the blade 3 region of the structure, could be due to qualitative or quantitative differences that modulate the otherwise conserved repression mechanisms in a context dependent fashion.

## Discussion

We previously proposed that the very high divergence in the central histone-binding repression domain might be responsible for the functional diversification of Tup11 and Tup12. The possibility of adaptive variation in this region is suggested by the fact that the two *S. pombe* proteins differ from each other in the central region to a greater extent than either of them does in relation to Tup1 proteins from a range of distantly related fungal species [18]. Phylogenetic analysis using sequences from different fission yeast species gives some support for this because Tup12 proteins tend to be more related to each other in the middle region than they are to Tup11 proteins. This is particularly true for comparison between the *S. pombe* and *S. octosporus* proteins, which both have full ability to complement CaCl_2_ stress related defects associated with *S. pombe* strains lacking Tup12. However, there is no equivalent tendency towards sequence conservation within the Tup11 group of proteins. Thus any adaptive changes within the middle region leading to Tup12-specific functions would have to be viewed in the light of the significant neutral variation that appears to be manifested by the Tup11 group of proteins. Importantly, replacing the Tup12 repressor domain with the equivalent region of Tup11 did not cause any noticeable effects on Tup12 specific functions under CaCl_2_ stress conditions. Thus the middle domain is not important for Tup12-specific function under these conditions. We cannot however, exclude the possibility that differences in the middle domain of Tup11 and Tup12 have a functional significance under other conditions that are not studied here. It is also possible that divergence in the middle domain has been of adaptive significance during evolution but that it no longer contributes to the genetic fitness of *S. pombe*.

Both the N-terminal and C-terminal domains show a higher degree of similarity within groups of different fission yeast Tup11 and Tup12 proteins, respectively, than for comparisons between Tup11 and Tup12 proteins. This is particularly true for *S. pombe* and *S. octosporus*, which both have a Tup12 that is fully active in the CaCl_2_ stress response of *S. pombe*. The C-terminal domain showed the strongest Tup11 and Tup12 specific conservation and our results clearly show that replacement of the Tup11 C-terminus with the corresponding region of Tup12 is sufficient to elicit Tup12-specific functionality that is indistinguishable from the intact Tup12 protein, at least in the context of CaCl_2_ stress. A predominant role of the C-terminal domain in Tup12 specificity would be consistent with the current views about Tup1 protein structure and function, whereby the N-terminus is primarily involved in interactions needed for co-repressor complex formation while the C-terminus is important for interaction with downstream factors involved in gene repression. Although this work has focused on the C-terminal WD40 domain, we have also studied a fusion protein where the N-terminal domain of Tup11 is replaced by the corresponding domain from Tup12. In this study the hybrid protein (Tup12-11-11) was fully able to rescue low-level CaCl_2_ sensitivity, which can be rescued by intact Tup11 or Tup12 proteins, but it was only weakly able to rescue high-level CaCl_2_, which requires Tup12-specific activity for rescue (data not shown). Unfortunately, we have not been able to measure the expression level of this protein (data not shown) and our data is thus inconclusive. It is possible that the Tup12 N-terminus contains only partial Tup12-specific activity since we know that sufficient protein is expressed to rescue low-level CaCl_2_ sensitivity but we cannot exclude the possibility that the lower Tup12-specific activity of the hybrid protein results from a lower expression level of this hybrid compared to the other expressed proteins.

We focused our attention on the C-terminal WD40 repeat domain for which a tertiary structure is available for the equivalent domain of Tup1 from *S. cerevisiae*. The location of divergent residues that consistently differ between Tup11 and Tup12 proteins is informative and useful for hypothesis building. For example, many mutations that interfere with Tup1 dependent genome-wide transcriptional regulation in *S. cerevisiae* [1], either at the level of corepressor recruitment or downstream steps, localize to the loop regions forming the top surface of the WD40 repeat structure. Thus, it was possible that differential requirement for Tup11 and Tup12 on subsets of genes might be due to distinct surface properties in this region. However, our results indicate that this region is completely devoid of significant differences between Tup11 and Tup12. Instead, the significant surface differences between Tup11 and Tup12 point mainly towards the third propeller blade of the WD40 repeat structure, revealing two distinct patterns on the side of the WD40 repeat modules of Tup11 and Tup12, respectively. Tup11 shares more identical residues with Tup1 in this region than does Tup12 (see residues shaded red compared to green in Fig 4), which is interesting, considering that Tup11 is generally less similar to Tup1 than is Tup12 (see Fig. 1). It is noteworthy that functional similarity between the Tup1 and Tup11 C-terminal domains *in vivo* has been demonstrated previously [11]. One possibility is that Tup11 and Tup12 might generally interact similarly with transcription factors and co-factors through the conserved top region of the structure while differing in overall affinity for some factors due to distinct interaction properties along the side of the WD40 repeat module, which might provide auxiliary interactions. In future work, it would clearly be relevant to search for protein interaction partners that show qualitative or quantitative differences in their interaction with Tup11 and Tup12 as well as to identify CaCl_2_ stress response genes for which the Tup12 C-terminal WD40 domain plays a specific regulatory role.

In conclusion, our present results show that the important determinants specifying Tup12-specific function during CaCl_2_ stress reside in the C-terminus of Tup12, and suggests that Tup12 specific surface properties in the third blade of the WD40 repeat domain propeller-like structure may play an important role in Tup12 specificity. Tup11 and Tup12 in fission yeasts thus provide an interesting system for studying the basis for functional specificity of WD40 repeat domains, that have a highly conserved overall structure.

## Materials and Methods

### Yeast strains and growth conditions

Unless otherwise stated, yeast strains (Table 1) were grown at 30°C in YES or, for selection of plasmids, synthetic minimal medium supplemented with the required amino acids. Solid media were supplemented with agar (2%). Strain FFB52 was constructed by PCR based replacement of the *leu^+^* cassette in strain JY741 *Δtup12* with a *kanMX* cassette. Complementation assays were carried out by spotting dilution series of logarithmically growing cells onto stress inducing and control plates, followed by 4–5 days incubation at 30°C.

**Table 1:** Strains used in this study.

Strain	Genotype	Reference
JY741	*h^−^ ura4-D18 leu1-32 ade6M216*	[11]
JY741 (*Δtup12*)	*Δtup12::LEU2^+^ h^−^ ura4-D18 leu1-32 ade6M216*	[11]
FFB52	*Δtup11::ura4^+^ Δtup12::kanMX h^−^ ura4-D18 leu1-32 ade6M216*	This study
yFS286	*Wild-type S. octosporus*, *haploid*	A. Smialowska
yFS275	*Wild-type S. japonicus*, *haploid*	NBRP^a^

a http://yeast.lab.nig.ac.jp/nig/index_en.html.

### Plasmids and cloning

*Escherichia coli* strain XL1-blue was used for cloning and amplification of plasmids.

Plasmids for expression of N-terminally FLAG-tagged full-length *S. pombe* Tup11 and Tup12 proteins were a generous gift from Dr. Simon Whitehall, University of Newcastle, UK. Constructs for expression of N-terminally FLAG-tagged S. pombe Tup11/12 hybrids were prepared by overlap extension PCR, using Expand High Fidelity System (Roche), and subsequently cloned into the BamHI site of pREP41-FLAG-N. Correct fusions of domain-encoding sequences were confirmed by PCR. Total RNA from logarithmically growing overnight cultures of wild-type *S. octosporus* and *S. japonicus* was prepared by hot phenol extraction, followed by further purification using a RNA Easy mini kit (Qiagen) and treatment with amplification grade DNase I (Invitrogen). Approximately 100 ng total RNA was used per 50 µl reaction consisting of the SuperScript III Platinum One-Step RT-PCR System (Invitrogen) and gene specific primers with added restriction sites. Platinum *Taq* Polymerase (Invitrogen) was used for –RT controls. *S. octosporus* and *S. japonicus tup11^+^* and *tup12^+^* cDNA was cloned into the BamHI site of pUC19 (Fermentas, #SD0411). *S. japonicus tup11^+^* cDNA was cloned into the BamHI site of pUC19 using BglII cleaved ends. Plasmids purified from five clones for each of the constructs were sequenced using M13 primers (Fermentas) and nested primers to obtain full coverage of both strands. Consensus cDNA sequences have the following accession numbers: GU253463, GU253464, GU253466 and GU253467. cDNA encoding *S. octosporus* Tup11 and Tup12 and *S. japonicus* Tup12 was subcloned into pREP42X (ATCC # 87607). *S. pombe tup12^+^* was sub-cloned from prep-FLAG-N-*tup12^+^* into pREP42X to serve as a control. Primer sequences are available upon request.

### Bioinformatics

Phylogenetic analysis of translated full-length protein sequences was performed using the neighbor-joining method implemented in the MEGA software package [20]. Comparison of protein domain sequences was performed by ClustalW alignment of domain regions in MacVector 6.5 using the BLOSUM62 matrix with a gap penalty of 10. ClustalX was used for alignment of C-terminal regions with secondary structure annotations, using a penalty function based on the known secondary structure of the *S. cerevisiae* Tup1 C-terminal domain [21]. Structure images were made in UCSF Chimera [22], using chain C of the crystal structure of the C-terminal domain of *S. cerevisiae* Tup1, PDB accession number 1ERJ [21].
